# Microsurgical treatment of breast cancer-related lymphedema under contrast-enhanced ultrasound guidance: a case report and literature review

**DOI:** 10.3389/fonc.2025.1677050

**Published:** 2025-11-05

**Authors:** Qiuchan Zhao, Xing Huang, Weizhang Chen, Yi Xiao, Jialing Zhang, Yujun Liu, Rongkang Liang, Zhongzeng Liang

**Affiliations:** ^1^ Department of Breast Surgery, Affiliated Hospital of Guangdong Medical University, Zhanjiang, Guangdong, China; ^2^ Department of Ultrasound, Affiliated Hospital of Guangdong Medical University, Zhanjiang, Guangdong, China; ^3^ Department of Hepatobiliary and Pancreatic Surgery, Affiliated Hospital of Guangdong Medical University, Zhanjiang, Guangdong, China

**Keywords:** breast cancer-related lymphedema, lymphaticovenous anastomosis, contrast-enhanced ultrasound, supermicrosurgery, functional lymphatic mapping

## Abstract

Lymphedema remains a chronic and challenging condition with limited curative options. Recent advances have expanded treatment strategies from comprehensive conservative management to microsurgical interventions, particularly lymphaticovenous anastomosis (LVA). LVA is a physiological surgical method in which functional lymphatic vessels are connected to nearby subdermal venules, enabling lymphatic fluid to bypass obstructed pathways and drain into the venous circulation. The success of LVA depends heavily on the accurate preoperative assessment and localization of functional lymphatic vessels. Contrast-enhanced ultrasound (CEUS) offers a valuable, non-invasive tool for identifying deep lymphatic channels, enabling dynamic evaluation of lymphatic contractility, peristalsis, and lymph flow. Furthermore, CEUS facilitates the identification of appropriately sized recipient veins, thereby reducing operative complexity and shortening surgical duration. We report a case of secondary upper limb lymphedema following breast cancer surgery, in which LVA was successfully performed under CEUS guidance using supermicrosurgical techniques.

## 1 Introduction

Approximately 10% of interstitial fluid in the human body is returned to the bloodstream via the lymphatic system Amore, Tapia, Mercado, Pattarone, Ciucci (1). Structural damage to the lymphatic network—caused by factors such as lymphatic pathway obstruction, lymph node dissection during cancer surgery, and recurrent infections—can lead to the development of lymphedema (2). At present, there is no definitive cure for lymphedema; treatment is primarily aimed at mitigating disease progression and alleviating symptoms. Conservative management typically includes complex decongestive therapy (CDT), which comprises compression bandaging, manual lymphatic drainage, physical therapy, and skin care (3). When non-surgical approaches fail to provide adequate relief, surgical interventions may be considered to restore functional lymphatic drainage. The advent of supermicrosurgery has significantly enhanced the surgical treatment of lymphedema. According to the consensus reached at the First European Supermicrosurgery Meeting in 2010, anastomoses involving vessels with diameters ranging from 0.3 mm to 0.8 mm are defined as supermicrosurgical procedures. Within this field, physiological lymphatic reconstruction—particularly LVA—has emerged as a key application and is now widely practiced (4).Lymphaticovenous anastomosis (LVA) is a microsurgical technique that diverts lymphatic flow by connecting intact lymphatic vessels directly to nearby venules, allowing lymph to bypass blockages and enter the venous system (5). Lymphovenous anastomosis (LVA), first described by K. Yamada as a technique to bypass damaged lymphatic pathways, is an effective treatment for lymphedema. By directly connecting impaired lymphatic vessels to adjacent veins, LVA creates an alternative drainage route that allows accumulated lymphatic fluid to be rerouted from swollen tissues back into the venous circulation. This physiological bypass helps restore lymphatic outflow, thereby reducing fluid buildup, swelling, tissue pressure, and stiffness associated with lymphedema (6, 7). Accurate preoperative assessment and precise localization of functional lymphatic vessels are critical for reducing operative time and improving the success rate of LVA. Indocyanine green (ICG) fluorescence lymphography is currently the most widely used modality for lymphatic mapping; however, it has notable limitations. ICG is restricted to visualizing superficial lymphatics (within approximately 1.5 cm of the skin surface),does not delineate adjacent venous structures, is contraindicated in iodine-allergic patients, and offers limited assessment of lymphatic function (8–10). In contrast, CEUS allows for real-time, dynamic evaluation of both superficial and deep lymphatic vessels, assessment of lymphatic contractility and flow, and identification of suitable recipient veins—all of which contribute to more effective surgical planning and execution. In this report, we present a case of secondary upper limb lymphedema following breast cancer surgery, successfully treated with CEUS-guided LVA utilizing supermicrosurgical techniques. This case highlights the clinical utility of CEUS in enhancing the precision of LVA and is accompanied by a review of current literature on the epidemiology, diagnosis, and management of breast cancer-related lymphedema.

## 2 Case report

A 74-year-old female patient underwent a right modified radical mastectomy for breast cancer at an outside hospital in 2000, followed by adjuvant chemoradiotherapy. Four years after surgery, she began to develop swelling of the right upper limb, which was not given attention. In 2021, she developed a chronic non-healing ulcer on the anterior chest wall accompanied by erythema, swelling, warmth, pain, and exudation. The diagnoses were: (1) chronic radiation-induced ulcer of the right chest wall; (2) multiple osteomyelitis involving the sternum and ribs. On April 7, 2022, she underwent an extensive debridement and resection of the right chest wall ulcer combined with a pedicled transverse rectus abdominis myocutaneous (TRAM) flap and free transverse rectus abdominis myocutaneous flap for one-stage chest wall reconstruction at our institution.

**Figure 1:**
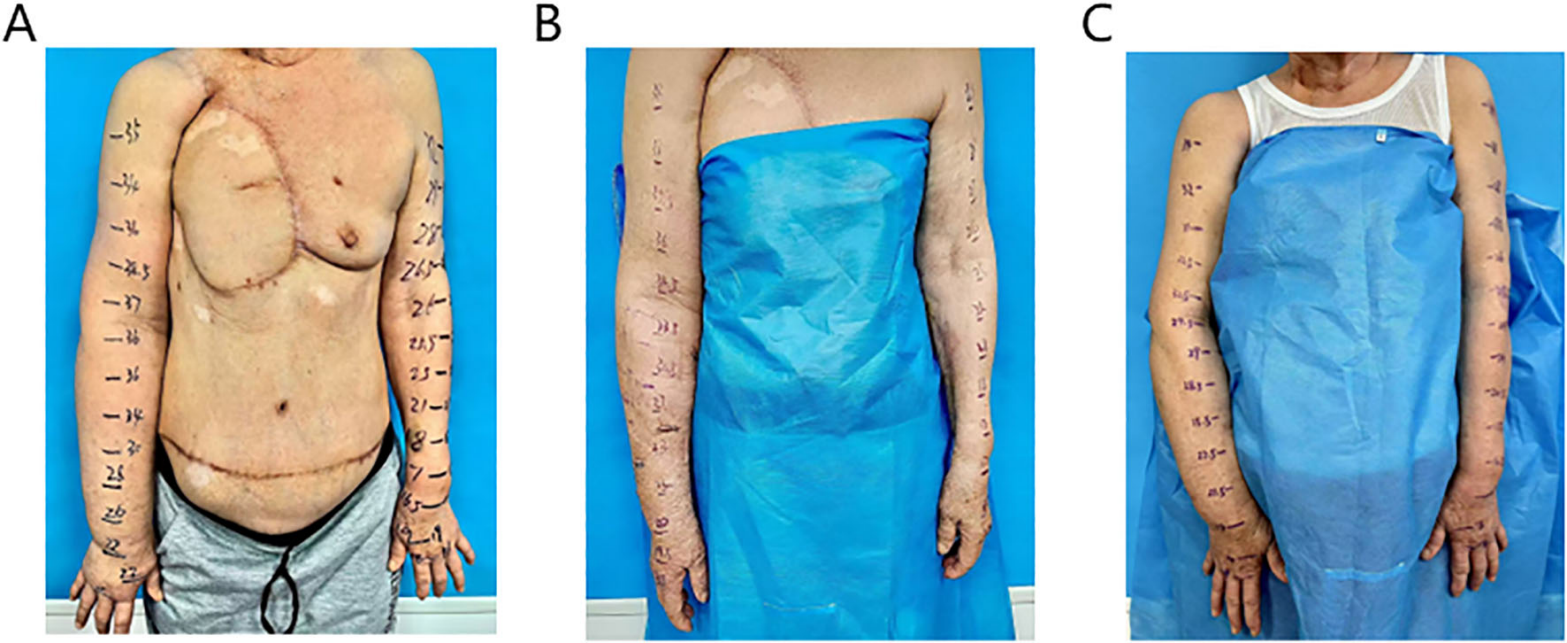
The preoperative condition of the affected limb showed significant swelling **(A)**, postoperative day 10, there was a marked reduction in edema in the right upper limb **(B)**, he five-month postoperative follow-up, the patient continued to show sustained improvement **(C)**.

During postoperative follow-up after chest wall reconstruction, physical examination revealed absence of the right breast, with a circumferential scar measuring approximately 20 cm × 10 cm. The left breast showed no deformity or abnormalities. But notably, the right upper limb exhibited significant swelling, with circumference markedly greater than that of the left upper limb (**Figure 1A**). After a period of conservative treatment without obvious improvement, the patient was scheduled to undergo LVA to alleviate upper limb lymphedema.

**Figure 2:**
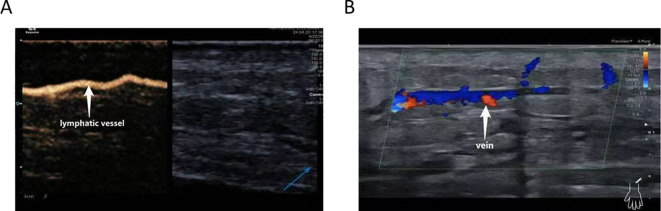
CEUS–assisted localization of functional lymphatic vessels **(A)** and target veins adjacent to lymphatic vessels **(B)**.

**Figure 3:**
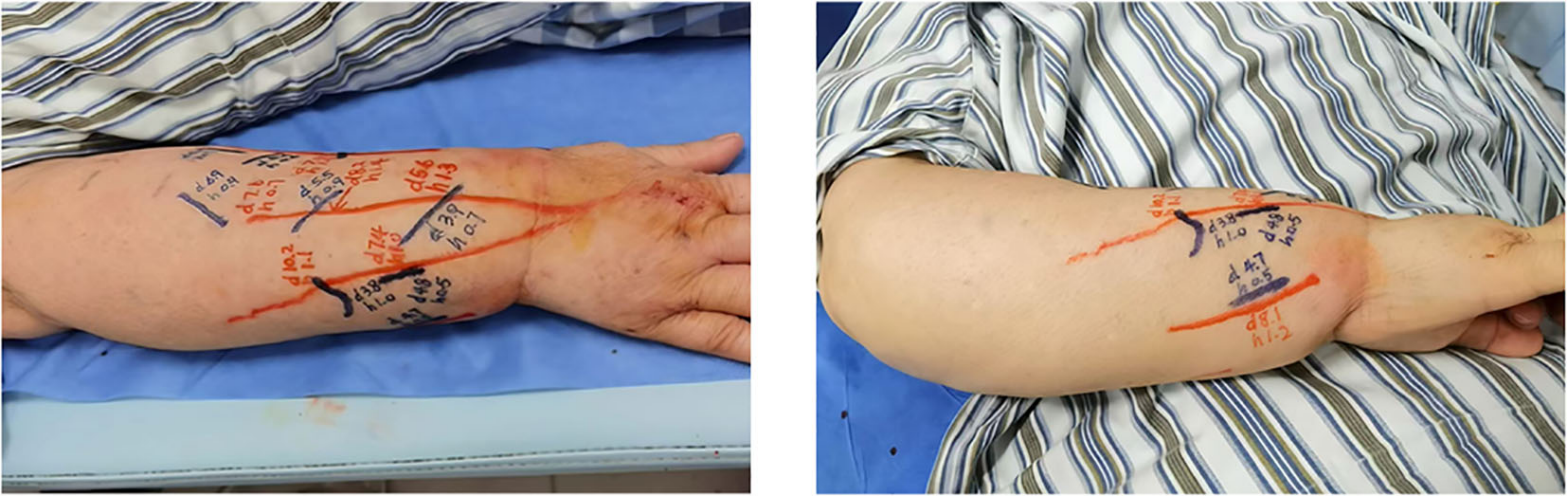
CEUS–assisted localization of lymphatic vessels and target veins in the right forearm.

**Figure 4:**
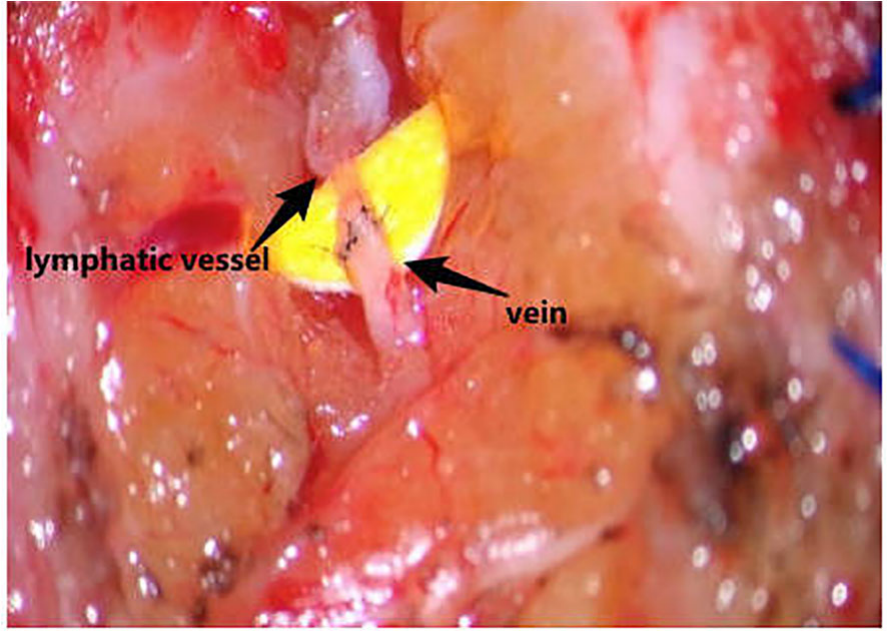
Traoperative anastomosis between the localized lymphatic vessel and the target vein.

CEUS was employed to precisely localize functional lymphatic vessels and target recipient veins (**Figure 2**) one day prior to surgery, followed by precise skin marking for intraoperative localization (**Figure 3**). On April 23, 2024, the patient underwent right upper limb lymphaticovenous anastomosis under general anesthesia. The surgical procedure entailed the following steps: (1) the patient was placed in the supine position with the affected limb abducted at 90°, followed by endotracheal intubation and standard sterile preparation; (2) an incision was made at the site of preoperatively identified lymphatic vessels and recipient veins near lymph node basins, with careful dissection to isolate the vessels; (3) end-to-end anastomosis of lymphatic vessels to veins was performed using 11–0 nylon sutures under microscopic magnification (**Figure 4**);(4) meticulous hemostasis was ensured, and the subcutaneous tissue and skin were closed in layers with running sutures; (5) postoperative management included compression bandaging with elastic wraps, skin care, and rehabilitation exercises as part of comprehensive decongestive therapy.

## 3 Results

Circumferential measurements were obtained for both upper limbs of this patient. Presenting data from both sides allows for a direct comparison, which is clinically relevant as it more objectively highlights the degree of swelling in the affected right upper limb. We therefore consider the inclusion of bilateral measurements helpful for illustrating the extent of lymphedema in this case.

Significant reduction in swelling of the right upper arm was observed 10 days postoperatively. Circumferential measurements were taken at 5 cm intervals, starting from the first web space of the right hand and the wrist crease, extending proximally to the shoulder. A marked decrease in circumference was noted at each measurement site (**Figure 1B**). 5 months post-surgery, repeat measurements demonstrated further improvement in limb circumference compared to the 10-day postoperative assessment (**Figure 1C**). Detailed numerical data are presented in the **Table 1**.

**Table 1:** Preoperative and postoperative circumference measurements at different sites of the patient’s right upper arm.

	First dorsal web space	Wrist crease	5 cm proximal to wrist crease	10 cm proximal to wrist crease	15 cm proximal to wrist crease	20 cm proximal to wrist crease	25 cm proximal to wrist crease	Cubital crease	5 cm proximal to cubital crease	10 cm proximal to cubital crease	15 cm proximal to cubital crease	20 cm proximal to cubital crease	25 cm proximal to cubital crease
Preoperative right upper arm circumference	22 cm	22 cm	26 cm	28 cm	30 cm	34 cm	36 cm	36 cm	37 cm	38.5 cm	36 cm	34 cm	35 cm
Right upper arm circumference at 10 days postoperatively	19.5 cm	20 cm	23 cm	23 cm	27 cm	30.5 cm	33.5 cm	34.5 cm	36 cm	35.5 cm	33 cm	34 cm	–
Right upper arm circumference at 5 months postoperatively	20 cm	19 cm	21.5 cm	22.5 cm	25.5 cm	28.5 cm	29 cm	29.5 cm	32.5 cm	32.5 cm	31 cm	32 cm	34 cm

## 4 Discussion on breast cancer-related lymphedema

### 4.1 Epidemiological characteristics and risk factors

The overall incidence of breast cancer-related lymphedema (BCRL) ranges from 6.7% to 62.5%, with approximately 75% of cases occurring within the first postoperative year and 80% within two years after surgery (11). This condition has a profound negative impact on patients’ quality of life. The wide variation in reported incidence is largely attributed to differences in surgical techniques, duration of follow-up, and diagnostic criteria. Temporally, the risk of BCRL persists for many years; some patients present with overt lymphedema symptoms even 10 years after surgery. Recent evidence-based studies have identified three major categories of risk factors for BCRL: (1)Treatment-related factors: Axillary lymph node dissection (ALND) is strongly associated with increased risk of BCRL, far exceeding the risk associated with sentinel lymph node biopsy (2). Radiation therapy, particularly irradiation of the supraclavicular and axillary regions, significantly increases the risk by inducing tissue fibrosis and lymphatic obstruction (12).(2)Patient-related factors: Obesity (body mass index [BMI] > 30 kg/m²) and hypertension have been identified as independent risk factors (13).(3)Metabolic indicators: A 2025 study demonstrated that elevated postoperative high-density lipoprotein (HDL) and triglyceride (TG) levels are significantly associated with increased risk of lymphedema, suggesting that lipid profile alterations may serve as early predictive biomarkers (14). Importantly, BCRL risk is cumulative over time. A study from Indiana University reported that, even among patients who underwent prophylactic immediate lymphatic reconstruction, the incidence of BCRL increased from 2.5% at < 12 months to 3.7% at 12–24 months, reaching 7.0% beyond 24 months postoperatively, underscoring the need for long-term surveillance, especially in high-risk populations (15).

### 4.2 Clinical manifestations and staging

BCRL is a progressive condition. In its early phase, symptoms are often subtle and may present as intermittent heaviness or tightness in the affected upper limb, particularly after physical activity. This is followed by persistent swelling, which typically begins in the dorsum of the hand and forearm and gradually extends proximally (16). Characteristic clinical signs include pitting edema, increased limb circumference, and progressive skin thickening and induration. In advanced stages, elephantiasis-like changes may develop, characterized by hyperkeratosis, papillomatosis, and recurrent episodes of lymphangitis (17).

According to the staging system of the International Society of Lymphology (ISL) (18), BCRL is classified into four stages: (1) Stage 0 (subclinical stage): Lymphatic transport function is already impaired; however, no clinically visible or measurable edema is present. This stage may persist for months or even years, during which microscopic alterations such as lymphatic vessel dilation and valvular incompetence have already occurred. Early intervention during this phase offers the greatest potential benefit. (2) Stage I (reversible stage): Limb elevation reduces edema, and pitting is evident upon palpation. At this stage, no significant fibrosis is present, making it the optimal window for conservative management. Histologically, inflammatory cell infiltration and collagen deposition may be observed. (3) Stage II (spontaneously irreversible stage): Limb elevation fails to resolve swelling, tissue firmness increases, and pitting gradually disappears. This stage is characterized by progressive fibrosis, with fibroblast proliferation and collagen cross-linking leading to tissue remodeling. Patients typically require compression garments to control limb volume. (4) Stage III (lymphostatic elephantiasis): The affected limb becomes markedly enlarged with severe skin changes, including hyperkeratosis and papillomatosis, accompanied by adipose deposition and chronic inflammation. Patients at this stage are prone to recurrent infections and irreversible functional impairment. Importantly, BCRL progression is influenced by a bidirectional pathophysiological loop: impaired lymphatic drainage promotes chronic tissue inflammation, while inflammation further accelerates fibrosis, creating a vicious cycle. Therefore, early interruption of this pathological process is crucial for optimal outcomes (19).

### 4.3 Diagnostic methods and evaluation criteria

The diagnosis of BCRL requires a comprehensive assessment integrating clinical presentation, objective measurements, and imaging studies, thereby establishing a multidimensional evaluation system.

#### 4.3.1 Clinical assessment methods

Physical examination remains the most fundamental diagnostic approach. In addition to assessing limb symmetry and cutaneous changes, circumferential measurement is the most commonly utilized quantitative method in clinical practice. Measurements are typically performed at 5 cm intervals, starting from the wrist crease and extending proximally to the shoulder. A difference in limb circumference ≥2 cm at the same anatomical level between the affected and contralateral limb is considered diagnostic. Alternatively, volumetric assessment using the water displacement method may be employed, with a >10% increase in limb volume on the affected side regarded as positive. The water displacement technique is based on Archimedes’ principle and provides accurate limb volume estimation but is labor-intensive and time-consuming (3). In recent years, novel technologies such as three-dimensional laser scanning and infrared optoelectronic volumetry have emerged, offering enhanced precision and convenience in limb volume measurement (20).

#### 4.3.2 Imaging-based assessment techniques

(1) Indocyanine Green Near-Infrared Fluorescence Lymphography (ICG-NIRF):

This technique currently represents the mainstream approach for evaluating lymphatic function. Following intravenous administration of indocyanine green (ICG), a near-infrared imaging system enables real-time visualization of lymphatic vessel morphology and drainage dynamics, allowing precise localization of functional lymphatic channels (21).

(2) Lymphoscintigraphy:

By injecting radiolabeled tracers, such as technetium-99m–labeled antimony sulfide colloid, lymphatic transport function can be quantitatively assessed. Semiquantitative parameters, including the uptake index and clearance rate, objectively reflect the degree of lymphatic dysfunction. Studies have demonstrated that lymphoscintigraphy achieves a sensitivity of up to 73% in detecting early-stage (Stage 0) lymphedema, outperforming conventional clinical examination (22).

(3) Bioimpedance Spectroscopy (BIS):

As a noninvasive method, BIS measures variations in the ratio of extracellular fluid to total body fluid, enabling detection of subclinical tissue fluid accumulation and providing early warning of impending lymphedema (23). A prospective study reported that BIS could predict lymphedema onset 6–12 months earlier than traditional circumferential measurements (24).

(4) Contrast-Enhanced Ultrasound:

CEUS is an emerging technique capable of delineating deep lymphatic vessels and providing a more comprehensive assessment of lymphatic function. It allows dynamic visualization of lymphatic vessel contractility, peristalsis, and lymph flow, as well as the identification of small veins anatomically paired with functional lymphatic channels. This capability substantially reduces surgical complexity and operative duration (25).

#### 4.3.3 Integrated diagnostic criteria

According to the 2023 ISL consensus, a diagnosis of breast cancer–related lymphedema (BCRL) requires fulfillment of at least two of the following criteria: (1) A circumferential difference of ≥2 cm between the affected and contralateral limb at the same anatomical level, persisting over two consecutive follow-up assessments. (2) A limb volume increase >10%, determined by water displacement or three-dimensional scanning. (3) Indocyanine green lymphography demonstrating lymphatic dilation, collateral pathway formation, or reflux abnormalities such as the “dermal backflow” pattern (“fountain sign”). (4) Lymphoscintigraphy showing a radiotracer retention time >60 minutes or a reduction in lymphatic uptake rate >30% (26).

### 4.4 Therapeutic strategies

The stepwise management of lymphedema follows the principle of “conservative therapy as the first-line approach, supplemented by surgical intervention when necessary.” Conservative treatment centers on CDT, which encompasses multilayer compression bandaging, manual lymphatic drainage, targeted functional exercises, and meticulous skin care. When conservative measures fail to achieve satisfactory outcomes, surgical reconstruction of functional lymphatic drainage pathways may be considered. Currently, the primary surgical options include lymphaticovenular anastomosis, vascularized lymph node transfer(VLNT), autologous lymphatic vessel transplantation, venous grafting, and omental flap transfer, all of which aim to restore or enhance lymphatic transport function (27, 28).

#### 4.4.1 Lymphaticovenular anastomosis

Lymphaticovenous anastomosis (LVA) techniques have progressively evolved since their initial description, with refinements aimed at reducing venous reflux and improving surgical efficacy. In 1969, Sedlácek (29) first attempted an anastomosis between the femoral lymphatic vessels and the great saphenous vein trunk. However, because of the high venous pressure in the great saphenous vein, there was a risk of retrograde blood flow into the lymphatic system. To overcome this limitation, subsequent studies proposed ligating tributaries of the great saphenous vein to reduce trunk venous pressure (30). With continuous advancements in microsurgical techniques, most surgeons now choose to perform anastomoses with small-caliber venules, which have lower intraluminal pressure (31).

Preoperative localization and functional assessment of lymphatic vessels are crucial for LVA. Currently, ICG near-infrared fluorescence lymphography is the reference standard for visualizing lymphatics during LVA (32). Nevertheless, ICG lymphography has notable limitations: it is unable to depict lymphatic vessels located deeper than 1–1.5 cm^9^, and in one recent study, it visualized target lymphatic vessels in only 40% of patients with lower-limb lymphedema (10). Moreover, ICG is contraindicated in patients with iodine allergy (8). Because ICG lymphography cannot visualize adjacent veins, additional imaging modalities are often required to identify the target venules for anastomosis, rendering the overall preoperative workflow relatively complex.

A 2022 retrospective study from the Mayo Clinic demonstrated that CEUS successfully identified functional lymphatic vessels in all enrolled breast cancer patients, including five individuals in whom ICG lymphography failed to detect lymphatics (33). Similarly, a 2023 study from the First Affiliated Hospital of Nanchang University reported that CEUS effectively visualized and accurately localized superficial lymphatic vessels. Compared with ICG lymphography, CEUS located lymphatic vessels with larger diameters and enabled precise measurement and localization of deeper lymphatic channels, significantly reducing operative time (34).

CEUS is a low-risk, convenient, cost-effective, and reproducible imaging technique. A commonly used contrast agent, SonoVue, composed of sulphur hexafluoride microbubbles, is typically injected subcutaneously into defined sites: in the upper limb, the interdigital spaces (1st–2nd and 4th–5th) and the palmar radial side of the wrist; and in the lower limb, the interdigital spaces (1st–2nd), medial and lateral malleolus, as well as the medial and lateral knee regions including the popliteal fossa. Each site generally receives 0.5 ml of the suspension, followed by gentle massage for 15–20 seconds (35). Although SonoVue does not contain iodine or other allergenic iodinated compounds and is not contraindicated in patients with iodine allergy, hypersensitivity reactions such as anaphylactic shock, skin erythema, and changes in blood pressure or heart rate have been reported (36). Subsequently, scanning is performed along the lymphatic drainage pathway to trace the enhanced lymphatic channels, identify accompanying veins, and mark their positions on the skin surface. CEUS provides several distinct advantages for localizing functional lymphatic vessels: (1) it enables clear visualization of lymphatic vessel trajectory, peristalsis, lumen diameter, and potential lymphatic leaks while tracing to the draining lymph nodes; (2) it has a tissue penetration depth of >2 cm; (3) it allows functional assessment of lymphatic valves—centripetal and rhythmic contrast movement indicates competent valves, whereas sluggish or retrograde movement suggests valvular insufficiency, and diffuse contrast distribution indicates lymphatic leakage. By combining functional dynamic assessment with simultaneous anatomical imaging, CEUS overcomes the depth limitations and contraindications associated with ICG lymphography, substantially reduces operative complexity and duration, and provides a highly cost-effective preoperative localization strategy for LVA.

With advances in portable ultrasound systems and AI-based segmentation algorithms, the integration of CEUS with LVA has the potential to evolve into a standardized surgical approach for lymphedema, significantly improving anastomotic efficiency while reducing the procedural learning curve (6). Furthermore, the widespread availability of CEUS devices facilitates the adoption of LVA in primary and secondary healthcare settings. Future integration of AI algorithms for automated identification of lymphatic pathways and optimal venous matching sites is expected to further shorten the learning curve (37).

Despite its clinical value in lymphedema assessment, CEUS exhibits notable limitations. The technique’s efficacy is highly dependent on precise intradermal injection; superficial or deep administration may compromise lymphatic uptake of contrast agents, resulting in suboptimal visualization. Furthermore, unlike ICG lymphography, CEUS fails to provide continuous mapping of lymphatic drainage pathways (38).

Immediate Lymphatic Reconstruction (ILR) refers to the microsurgical creation of lymphatic–venous anastomoses at the time of ALND. The team at Indiana University pioneered this approach, in which transected lymphatic vessels are anastomosed to adjacent venules intraoperatively, thereby reestablishing lymphatic drainage pathways. In a cohort of 172 patients with a median follow-up of 23.1 months, the cumulative incidence of BCRL was only 7.0% in the ILR group, significantly lower than 23.6% observed in the conventional ALND group (p < 0.05) (15). The procedure required a median of one anastomosis and increased operative time by only 35 minutes, with a low complication rate (<5%) and favorable cost-effectiveness, demonstrating its clinical feasibility and preventive potential.

Recent clinical evidence supports the preventive role of ILR in breast cancer-related lymphedema (BCRL). An interim analysis of a multicenter randomized controlled trial showed a markedly lower incidence of BCRL in the ILR group (9.5%) compared with controls (32%) after 12–24 months of follow-up, alongside improvements in bioimpedance, elastic sleeve use, ICG lymphography, and quality of life (39). Similarly, a meta-analysis of nine prospective studies (n = 791) reported pooled BCRL rates of 9% in patients receiving ILR versus 29% without ILR, further supporting its clinical benefit (40).

#### 4.4.2 Vascularized lymph node transfer

For patients with advanced BCRL or those in whom LVA has failed, VLNT offers an innovative therapeutic approach. In 1982, Clodius and colleagues (41)first attempted transferring inguinal lymph nodes from the unaffected limb to the affected limb, providing a novel concept of anatomical reconstruction for lower-limb lymphedema. In 2006, Becker et al (42)transplanted inguinal lymph nodes into the axilla or elbow of 24 patients with refractory upper-limb lymphedema. A five-year follow-up demonstrated that 75% (18/24) of patients experienced significant limb volume reduction, with complete resolution of recurrent cellulitis, thus establishing the feasibility of the procedure. In 2008, Lin et al (43)introduced the innovative technique of wrist-level lymph node transfer, based on the anatomical stability of the vasculature and minimal scarring in this region, as well as the natural accumulation of lymphatic fluid in the wrist due to gravity. All 13 patients undergoing this procedure achieved significant clinical improvement. Furthermore, Cheng et al (44)reported, in 2012, the transfer of submandibular lymph nodes to the ankle region for the treatment of lower-limb lymphedema. Evidence from recent studies (45)indicates that transferring a vascularized supraclavicular lymph node flap to the dorsum of the foot yields favorable therapeutic outcomes with a low complication rate. These advancements underscore the transition toward precise anatomical recipient-site selection, heralding a new era of targeted reconstructive lymphatic surgery.

#### 4.4.3 Lymphatic vessel transplantation

Baumeister et al (46) first reported the use of autologous lymphatic vessel transplantation for lower-limb lymphedema in 1981. The surgical principle involved harvesting lymphatic vessels from the unaffected limb and creating a subcutaneous tunnel through which the graft was implanted into the affected limb, with both ends anastomosed respectively to lymphatic vessels in the edematous and normal regions. Postoperative lymphangiography confirmed patency of the transplanted lymphatic channels, and long-term follow-up demonstrated significant edema reduction in some patients. However, this technique has three major limitations: (1) donor-site morbidity including iatrogenic lymphedema due to injury of the lymphatic system on the healthy side; (2) relatively long donor incisions, adversely impacting aesthetics and patient psychology; (3) high intraoperative failure rates in advanced cases due to fibrosis of recipient lymphatic vessels. Consequently, lymphatic vessel transplantation is rarely performed clinically today.

#### 4.4.4 Venous grafting

Venous grafting shares a similar core principle with autologous lymphatic vessel transplantation, aiming to reconstruct the lymphatic circulation by transplanting healthy vascular or lymphoid tissue to relieve lymphatic stasis. The standardized procedure involves harvesting a segment of vein with good patency and elasticity from the unaffected limb, which is then microsurgically anastomosed proximally and distally to the congested and normal lymphatic vessels, respectively. Precise and stable vascular anastomoses are ensured to prevent postoperative complications such as leakage or thrombosis. Campisi et al (47)introduced a novel technique involving harvesting a 7–15 cm segment of vein from the surgical site on the affected limb, connecting multiple lymphatic vessels to one end of the vein to increase lymphatic flow and prevent venous occlusion. Venous valves guide unidirectional lymphatic flow. Compared to lymphatic vessel transplantation, venous grafting offers advantages including larger vessel diameter and thicker, more resilient vessel walls, which reduce microsurgical complexity. Additionally, veins possess favorable elasticity and contractility, better accommodating lymphatic return demands. However, venous grafting also entails risks and challenges; postoperative venous reflux may induce lymphangitis, and endothelial functional differences between veins and lymphatics require a period of adaptation before optimal lymphatic drainage function is restored.

#### 4.4.5 Omental transfer

The greater momentum is a vital intra-abdominal structure rich in lymphatic and vascular networks, playing critical roles in immune response, fat storage, and inflammation modulation. Based on its anatomical characteristics, Goldsmith et al. (48) proposed pedicled or free omental flap transfer based on the gastroepiploic vessels as an innovative approach to alleviate lymphatic stasis. However, the clinical application of this technique remains challenging due to the complexity and inherent risks of the procedure, including graft rejection, intra-abdominal adhesions, bowel obstruction, and potential need for reoperation. Given these limitations, despite its promising therapeutic potential, omental transfer requires careful patient selection and risk-benefit evaluation. Advances in microsurgical techniques are essential to optimize surgical safety and establish standardized treatment protocols.

### 4.5 Prospects of LVA surgery assisted by CEUS for preoperative mapping

In the case reported herein, CEUS-guided LVA resulted in sustained symptomatic improvement without recurrence of lymphedema. Based on the accumulating evidence and experimental validation, CEUS-based functional lymphatic vessel mapping represents an effective strategy to optimize clinical outcomes of LVA and warrants broader application as a precision surgical intervention for lymphedema.

## Data Availability

The original contributions presented in the study are included in the article/Supplementary Material. Further inquiries can be directed to the corresponding author.
